# Exercise as a modulator of gut microbiota for improvement of sleep quality: a narrative review

**DOI:** 10.3389/fnins.2025.1639099

**Published:** 2025-11-17

**Authors:** Bin Zhao, Jinxiang Sun, Lijuan Xiang, Zhanguo Su

**Affiliations:** 1 Department of Physical Education Teaching, Lanzhou University of Finance and Economics, Lanzhou, Gansu, China; 2 Department of Sport Convergence, Namseoul University, Cheonan, Republic of Korea; 3 Chongqing Preschool Education College, Chongqing, China; 4 Faculty of Physical Education, Huainan Normal University, Huainan, Anhui, China; 5 International College, Krirk University, Bangkok, Thailand

**Keywords:** sleep, gut microbiome, dysbiosis, exercise, physical activity

## Abstract

Sleep quality is a cornerstone of physical and mental well-being, yet millions of individuals worldwide suffer from chronic sleep disturbances. Recent developments in microbiome research have shown that the microbiota–gut–brain axis regulates sleep by two-way communication between the gut and brain. Exercise is well-documented for its beneficial impact on sleep, yet emerging evidence indicates that one mechanism by which it achieves this may involve the modulation of gut microbiota. This narrative review examines the developing triadic relationship among exercise, gut microbiota, and sleep. It examines how exercise influences microbial diversity, the production of sleep-related metabolites like serotonin, GABA, and SCFAs, and immune responses that collectively shape sleep architecture. Additionally, the review highlights physiological factors influenced by exercise—such as gut motility, intestinal barrier function, and bile acid metabolism—that may impact the gut ecosystem and, in turn, sleep. Although encouraging results, deficiencies persist in our comprehension of the specific mechanisms connecting these domains. This review underscores the need for interdisciplinary research and suggests that targeting the gut microbiota via customized exercise interventions presents a promising, non-pharmacological strategy for enhancing sleep quality.

## 1 Introduction

A basic biological process necessary for preserving physical, cognitive, and emotional health is sleep (Baranwal et al., 2023). Poor sleep quality is being more and more acknowledged as a significant public health issue since it affects metabolic dysregulation and higher CDV risk along compromised immune function and cognitive decline (Lim et al., 2023; Kavousi et al., 2025; Sewell et al., 2021; Wang et al., 2022a). Though important, sleep problems are common and impact millions of people all around. Increasing amounts of studies have focused on the intricate, two-way interactions between physiological systems affecting sleep, including the CNS, immune activity, endocrine function, and more recently, the gut flora (Han et al., 2022; Gil-Hernández et al., 2023; Shen et al., 2023; Akset et al., 2023; Zhou et al., 2024; Wei et al., 2023).

The collection of organisms that live in the GI tract, known as the gut microbiota, is becoming an increasingly important component of the host’s overall health. The gut microbial ecosystem influences the CNS through endocrine, neural, metabolic, and immunological pathways (Ojeda et al., 2021; Doroszkiewicz et al., 2021). Growing proof now points to the gut microbiota’s ability to affect sleep architecture and quality by altering neurotransmitter synthesis (e.g., serotonin, GABA), systemic inflammation, circadian rhythm control, and metabolic homeostasis (Han et al., 2022; Wang et al., 2022b). Sleep problems—like restless leg syndrome, insomnia, and sleep apnea —is correlated to disturbances in the gut microbiome such as decreased diversity or dysbiosis (Neroni et al., 2021).

At the same time, many people have come to recognize exercise as a non-pharmacological treatment to enhance sleep quality. Consistent physical activity enhances sleep through various mechanisms including thermoregulatory changes, stress reduction, better mood, and strengthened circadian alignment (Xie et al., 2021). More lately, focus has shifted to the potential that changes in the gut flora could help to mediate some of exercise’s positive impact on sleep (Min et al., 2024). Exercise alters gut microbial diversity in humans and animals, according to many studies, raises good bacterial counts, and improves microbial metabolic output—qualities that could then affect sleep control (Aragón-Vela et al., 2021; Aya et al., 2023).

Though these encouraging findings point to a triadic interaction between exercise, gut flora, and sleep quality that is still under-researched in an integrated way. Although there are studies looking at how exercise affects the microbiome and how the microbiome affects sleep, few have combined these results to determine whether gut microbiota changes brought on by exercise act as a mechanism for sleep enhancement. Most of the studies now in existence either isolate these variables or neglect to define causal pathways and mediating factors including exercise duration, type, and intensity of exercise. Individual variation in gut microbiota composition and response to exercise also makes it more difficult to draw universal conclusions (Kelsey et al., 2021; Wu et al., 2021).

This gap in the literature highlights a critical need. While the individual links between exercise and microbiota, and microbiota and sleep, are being established, the field lacks an integrated synthesis that moves beyond descriptive summaries. Simply acknowledging this triadic relationship is insufficient when the underlying mechanisms of interaction remain poorly defined.

**Figure 1:**
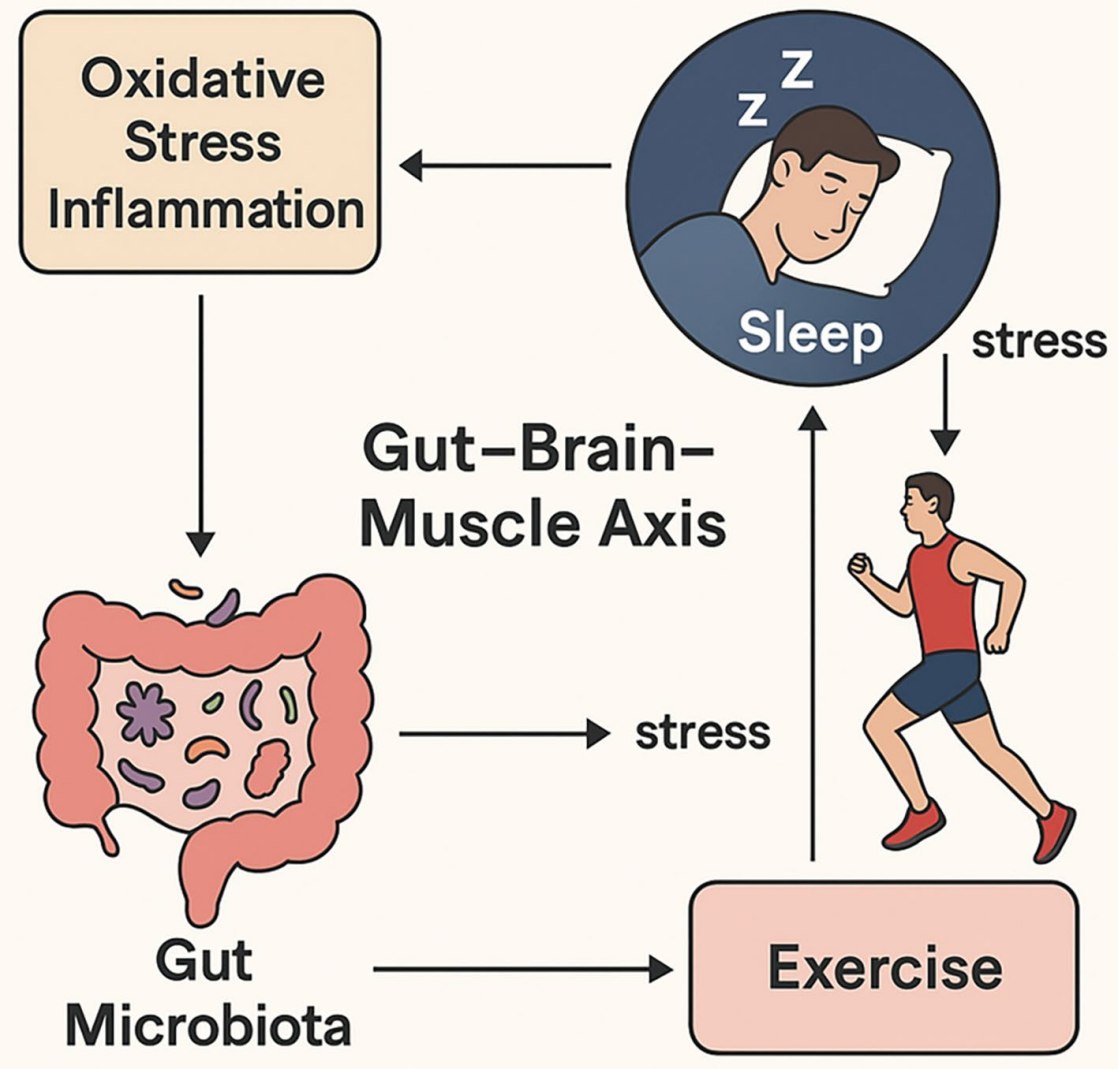
Schematic depiction of the interrelated functions of exercise, gut microbiota, sleep, inflammation, and oxidative stress within the gut–brain–muscle axis. The diagram illustrates how regular physical activity reduces oxidative stress and systemic inflammation, supporting beneficial gut microbial growth and enhancing sleep quality. Conversely, sleep disorders like OSAS can disrupt gut barrier function, promote bacterial translocation, and increase inflammation, impairing both sleep and exercise recovery. Chronic stress and elevated cortisol further exacerbate this cycle. Exercise acts as a modulatory force that can break this cycle, stabilizing gut health, improving muscle recovery, and restoring healthy sleep patterns.

Therefore, this review provides a critical, integrative analysis of the exercise-microbiota-sleep axis. We move beyond a sequential presentation of findings to structure our review around the shared biological pathways—including inflammatory signaling, microbial metabolite production (e.g., SCFAs), and neuroendocrine regulation—that form the communication network between these three domains. We will critically appraise the current evidence, highlighting key controversies and methodological challenges that impede progress. By synthesizing these data, we propose a cohesive conceptual framework, visually summarized in Figure 1, that models the feedback loops within this axis, offering a clear roadmap and testable hypotheses to guide future interdisciplinary research.

## 2 Literature search strategy

The literature for this narrative review was identified through a comprehensive search of the PubMed (MEDLINE), Scopus, and Google Scholar electronic databases for articles published between January 2010 and September 2025. A supplementary search of reference lists from relevant articles was also conducted to identify foundational studies published prior to this period. The search strategy combined keywords from three core domains using Boolean operators (AND/OR): (1) Exercise domain: ‘exercise’, ‘physical activity’, ‘training’, ‘endurance’; (2) Microbiota domain: ‘gut microbiota’, ‘gut microbiome’, ‘dysbiosis’, ‘intestinal flora’; and (3) Sleep domain: ‘sleep’, ‘insomnia’, ‘sleep quality’, ‘sleep architecture’, ‘circadian rhythm’.

We included peer-reviewed original research (both human and animal models) and review articles published in the English language. The primary inclusion criterion was a focus on the interactions between at least two of the three core topics of the review. Case reports, editorials, conference abstracts, and non-peer-reviewed literature were excluded. The final selection of articles was based on their relevance to elucidating the mechanistic links within the exercise-microbiota-sleep axis, with a preference for studies that provided insight into the physiological pathways discussed. With this foundation, we will first outline the fundamental, bidirectional relationship between the gut microbiota and sleep.

## 3 The gut-microbiota-sleep axis: a bidirectional relationship

### 3.1 Gut—brain axis

**Figure 2:**
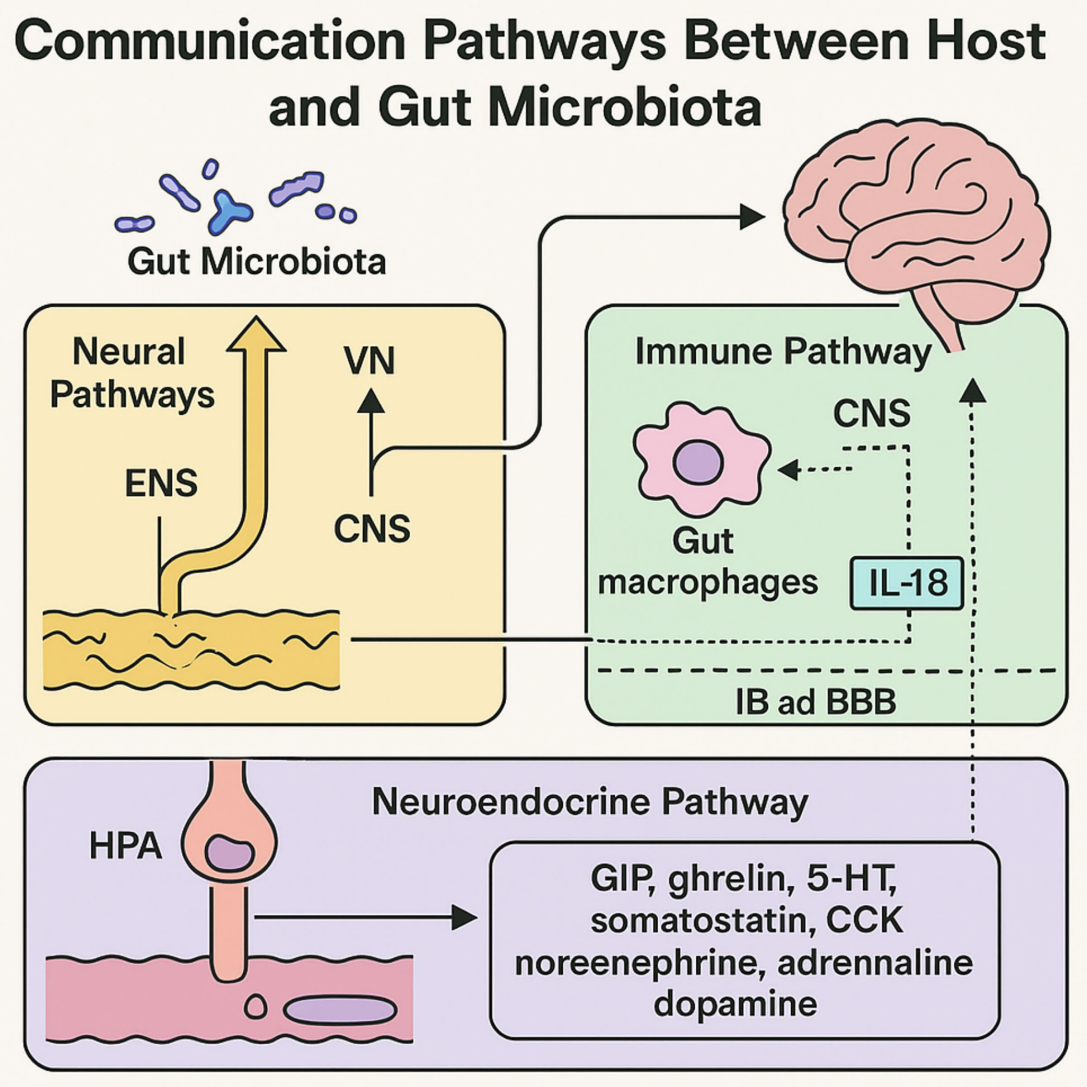
Illustration of interaction pathways between the gut microbiota and host within the gut-brain axis. The diagram illustrates three primary pathways: (1) Neural pathway, including the ENS, vagus nerve (VN), and CNS, which facilitate bidirectional communication and sensory integration through microbial metabolites and neurotransmitters; (2) Immune pathway, highlighting the function of gut immune cells, IB, and BBB in maintaining homeostasis and mediating inflammatory responses; and (3) Neuroendocrine pathway, centered on the HPA axis and enteroendocrine cells, which regulate stress responses and secrete key neurotransmitters like6 serotonin, norepinephrine, and dopamine. The interaction of these systems shows how gut microbiota affects brain function, emotion, and behavior.

Clinically, the brain and gut are generally regarded as two separate systems in terms of function and anatomy, with communication between them occurring through three mechanisms: the immune, neural and neuroendocrine pathways (Lu et al., 2024) (Figure 2).

The nervous system has a significant impact on the process by which the microbiota in the gut and the brain begin to communicate with one another (Geng et al., 2022). The intestinal submucosa and muscularis propria comprise neurons and glial cells (Pawolski and Schmidt, 2020). Apart from the CNS, the ENS regulates gastrointestinal function and physiology autonomously. It may also engage the CNS through neuroendocrine pathways, microbiota metabolites, and the vagus nerve (VN) (Drokhlyansky et al., 2020; Spencer and Hu, 2020). Compounds produced by gut microbiota and cells can stimulate afferent neurons in the ENS, leading to modifications in electrical activity in associated brain regions via the VN (Waise et al., 2018). This direct relationship indicates a long-term, bidirectional interaction between the ENS and the gut microbiota. On the other hand, the microbiota is essential for the development and growth of the ENS, as well as for preserving its functional and structural integrity. The ENS provides the microbiota with a living environment that is suitable for their existence (Obata and Pachnis, 2016; Joly et al., 2021). Changes in length, motility, permeability, and neuronal loss in the intramuscular and submucosal plexus of the ileum and proximal colon may occur in the murine GI tract and ENS as a result of gut microbiota depletion. LPSs and SCFAs originating from the gut microbiota can alleviate this deficiency (Vicentini et al., 2021). Releasing serotonin and activating 5-HT4 receptors facilitates the maturation of the adult ENS. Intestinal microbiota synthesize serotonin, which modifies enteric nervous system neuroanatomy and accelerates gastrointestinal transit (Qiu et al., 2023).

VN connects the CNS and the ENS as a bridge and substances from gut microbiota access the CNS in this way (Dicks, 2022; Heiss and Olofsson, 2019). Sends gut luminal signals from VN to dorsal hippocampus glutamatergic neurons in milliseconds, intestinal glutamate metabolism allows gut stimuli to be sensed with synapse-like temporal and topographical resolution (Kaelberer et al., 2018; Suarez et al., 2018). The right vagal ganglion of the intestine optically stimulates glutamatergic neurons in the substantia nigra, releasing dopamine. This regulates taste and boosts reward neurons (Han et al., 2018). Vagal ganglion neurons not only transmit signals from the ENS to the CNS but also innervate intestinal villi, regulate intestinal motility, and detect nutrients. Vagal-mediated gastrointestinal hormone responses are essential for regulating glucose homeostasis and eating behaviors (Williams et al., 2016).

In healthy guts, immune function-related gut macrophages control ENS differentiation in infancy and maintain normal neural function in adulthood (Delfini et al., 2022). Immune-related gut macrophages can be converted to protective phenotypes to prevent ENS neuron loss and intestinal motility issues after gut infection. ENS affects gut immunity by mediating immune responses. Studies have shown that gut neurons use IL-18 signals to regulate the gut immune barrier, which greatly affects the mucosal barrier and bacterial clearance (Ahrends et al., 2021). A CD14-dependent mechanism of action, the “gut microbiota-immune barrier homeostasis,” shields the brain from damage connected to inflammation (Hoyles et al., 2018). Furthermore, the “gut microbiota-immune barrier homeostasis” has a significant impact on the manner in which chromaffin cells located in the intestinal epithelium secrete 5-hydroxytryptamine (Wei et al., 2022). This 5-HT regulates gut-brain communication in peripheral circulation to affect emotions and visceral pain (Bayrer et al., 2023).

The intestinal barrier (IB) and BBB govern host-gut communication signals, metabolites, the brain-gut axis, and the relationship with gut microbiota (Welcome, 2019; Martens et al., 2018; Pellegrini et al., 2023; Wang et al., 2020). Microbiota metabolism may lower IB tight-junction protein levels, compromising IB function (Geng et al., 2020) and more BBB permeability (Braniste et al., 2014). Erroneous translocation and colonization of pathogenic bacteria causes neuroinflammation and cognitive decline after barrier damage (Li B. et al., 2019; Jansma et al., 2021; Zhao et al., 2023). Researchers found that gut microbiota dysbiosis in constipated mice increases IB and BBB permeability and inflammation. This disrupts helper and regulatory T cell expression in the nervous system, causing autoimmune encephalomyelitis-related problems (Lin et al., 2021). To protect brain tissue from harmful chemicals, the BBB regulates chemical transfer between brain parenchyma and peripheral blood. It protects the CNS from circulatory effects (Knox et al., 2022).

The hypothalamic paraventricular nucleus regulates responses from the sympathetic, parasympathetic, immune, metabolic, and CNS as a traditional neuroendocrine ring. Additionally, it is responsible for the secretion of the neuropeptide CRF. The HPA axis is responsible for the communication that occurs in both directions between the gut and the brain. This communication greatly affects health. Early research suggests that the endocrine pathway may connect the microbial gut and the brain during infancy, and that gut flora may affect HPA axis development. Both of these hypotheses have been supported by the findings of additional studies. Both of these hypotheses are supported by the findings of further research (Ge et al., 2021; Rosin et al., 2021). Probiotic therapy may diminish HPA axis hyperactivity, modulate gut microbiota composition, and substantially alleviate stress-induced anxiety behaviors (Tian et al., 2021). Dysbiosis within the internal environment stimulates the pituitary gland, resulting in a cascade amplification effect. This stimulates cortisol (CORT) production in the adrenal glands via adrenocorticotropic hormone. A negative feedback loop between the hippocampus, hypothalamus, and pituitary gland results (Lightman et al., 2020). Disrupted CORT secretion affects adrenocorticotropic hormone response to brain neurons, plasma glucocorticoid levels, HPA axis circadian rhythm, and CRF.

Gut neurotransmitters are detected by the host brain. Neurotransmission between the CNS and gut bacteria improves gut microbiota adaptation in the GI tract (Fung et al., 2019; Zhong et al., 2023; Kasarello et al., 2023). Enteroendocrine cells are the ones mostly in charge of generating hormones including neurotransmitters in the gut (Bellono et al., 2017; Yoo and Mazmanian, 2017). The most important enteroendocrine hormones were identified as ghrelin, 5-HT, cholecystokinin, gastric inhibitory peptide, and somatostatin, according to the time-resolved single-cell transcriptional map. These cells’ hormone synthesis reaction is fascinating and evolving. Aging gut epithelial cells produce several hormone (Okaty et al., 2019; Gehart et al., 2019). ECs release most peripheral 5-HT to the lumen, platelets, and mucosa. This activation promotes gut-CNS communication (Dodds et al., 2022; Yano et al., 2015; Agus et al., 2018). 5-HT is quite important in controlling emotions, hunger, sleep, and other bodily physiological processes. The 5-HT in the gut enters the bloodstream and is progressively carried to several regions of the CNS, including amygdala, hypothalamus, and cerebral cortex, therefore affecting their operation (Sharp and Barnes, 2020). Immune and gut epithelial cells interact with 5-HT to affect bowel movement and IB permeability (Sugisawa et al., 2020). In the gut, tyrosine produces DA, NE, and adrenaline. Gut flora may convert tyrosine into 4EPS, according to recent studies. This substance can cross the BBB and reduce brain myelinated oligodendrocyte development, causing anxiety. The neuroactive bacterial molecule 4EPS affects animal brain activity and sophisticated behavior, even though it is not a neurotransmitter (Needham et al., 2022). Memory problems, cognitive decline, and depressive behavior may be related to gut microbiota metabolites and neurotransmitters (Yang et al., 2021; Li et al., 2022; Zhao T. et al., 2022). Adsorbents were developed by the researchers in order to selectively capture small molecules of aromatic or phenolic compounds in the digestive tract. Changing neurotransmitter levels reduces gut microbial metabolite-induced anxiety in mice (Stewart Campbell et al., 2022). DA and other neurotransmitters like GABA also influence gut flora, affecting behavioral, cognitive, and emotional functions.

### 3.2 Gut microbiota and sleep

**Figure 3:**
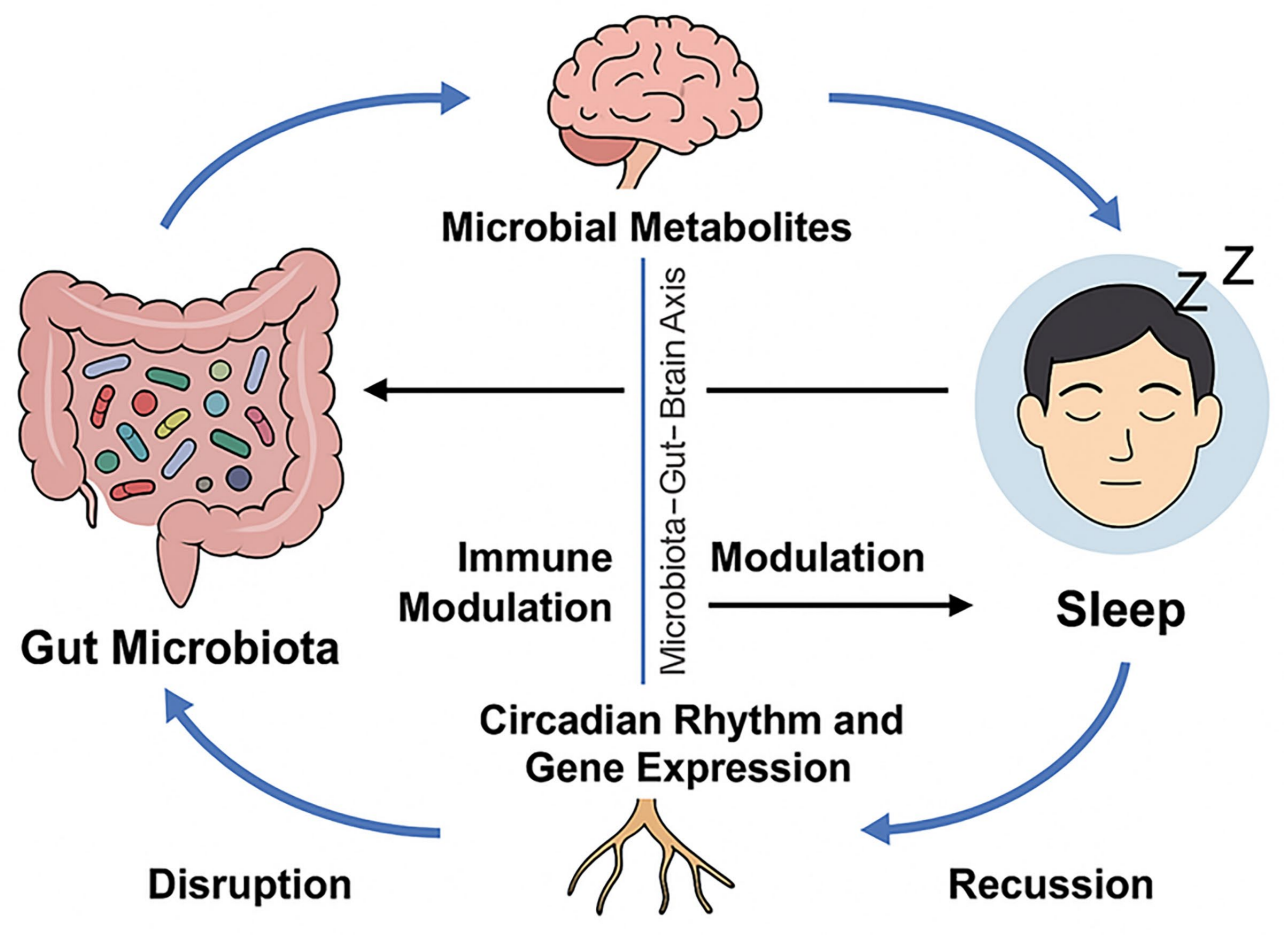
The relationship guts microbiota and between sleep is bidirectional, mediated through the microbiota–gut–brain axis. Neurotransmitter and SCFAs precursors from microbes affect sleep via neural and hormonal mechanisms. The gut microbiota influences sleep architecture through the modulation of immune responses. Sleep and circadian rhythms affect microbial composition and gene expression, demonstrating a dynamic, reciprocal relationship.

The complex and dynamic interaction between gut sleep and microbiota, marked by potential bidirectional effects, represents a significant focus of contemporary scientific inquiry. Recent research demonstrates that the microbiota-gut-brain axis regulates sleep patterns directly and indirectly, affecting sleep disorder etiology and progression. Studies demonstrate that sleep disorders modify gut microbiota composition, while sleep deprivation affects activity levels. Microbial metabolites affect sleep through the gut–brain axis, one of three main factors. The microbiota influence sleep regulation through modifications of immune responses. Sleep and gene expression dynamics affect gut microbiota composition (Lin et al., 2024) (Figure 3).

Gut microbiota metabolites mediate the microbiota–gut–brain axis and significantly affect sleep physiology. Through mechanisms involving neurotransmitter modulation, immune signaling, and circadian rhythm synchronization, these metabolites influence both sleep quality and architecture. Disruptions in the production or balance of these compounds are increasingly associated with sleep disturbances and inflammation. Table 1 summarizes the key gut-derived metabolites, their specific impacts on sleep regulation, and the corresponding evidence from recent studies.

**Table 1:** Impact of gut microbiota metabolites on sleep.

Metabolite	Impact on sleep	Key references
Serotonin (5-HT)	Regulates mood and sleep via gut-brain axis; increased by gut bacteria like Clostridiaceae and Turicibacteraceae; vagus nerve plays a key role.	Yao et al. (2021), Valdés-Cruz et al. (2002), McVey Neufeld et al. (2019), Fung et al. (2019), Li H. et al. (2019), Hata et al. (2017), Bacqué-Cazenave et al. (2020)
Histamine	Regulates intestinal and brain functions; produced by specific bacteria; H1 receptor antagonists explored for insomnia treatment.	Zisapel (2015), Barcik et al. (2016); Haas et al. (2008)
SCFAs	Produced by LAB and others; maintains gut barrier, reduces inflammation; depletion linked to sleep disruption and inflammation.	Wang et al. (2021), Yang et al. (2023), Szentirmai et al. (2019), Gao et al. (2023), Heath et al. (2020), Koh et al. (2016), Kieliszek et al. (2021)
Lipopolysaccharide (LPS)	LPS can induce non-REM sleep via IL-6; crosses BBB and activates microglia via TLR4, causing inflammation that impairs sleep.	Yang et al. (2023), Zhang et al. (2022), Lively and Schlichter (2018), Liu et al. (2021), Morrow and Opp (2005)
Other metabolites (e.g., GABA, melatonin, dopamine)	Melatonin restores gut flora and reduces inflammation after sleep loss; GABA-producing probiotics improve gut integrity and sleep.	Zhao N. et al. (2022), Gao et al. (2019), Wang et al. (2023)

The intestinal barrier must be intact to prevent pathogenic bacteria and their metabolites from entering the bloodstream. This, in turn, reduces the likelihood of infections and inflammation throughout the body. It also serves as a reservoir for macrophages, natural killer cells, dendritic cells, and other immune cells. The interaction between these cells and microorganisms in the gut is significant. This interaction develops and distinguishes immune cells to sustain immune homeostasis and the microbial ecosystem of the gut (Hand et al., 2016). Immune system activity regulates sleep patterns, and sleep quality affects immune system function and response (Besedovsky et al., 2019). This delicate balance emphasizes the synergy of gut health, immune competence, and sleep in preserving general resilience and health.

**Figure 4:**
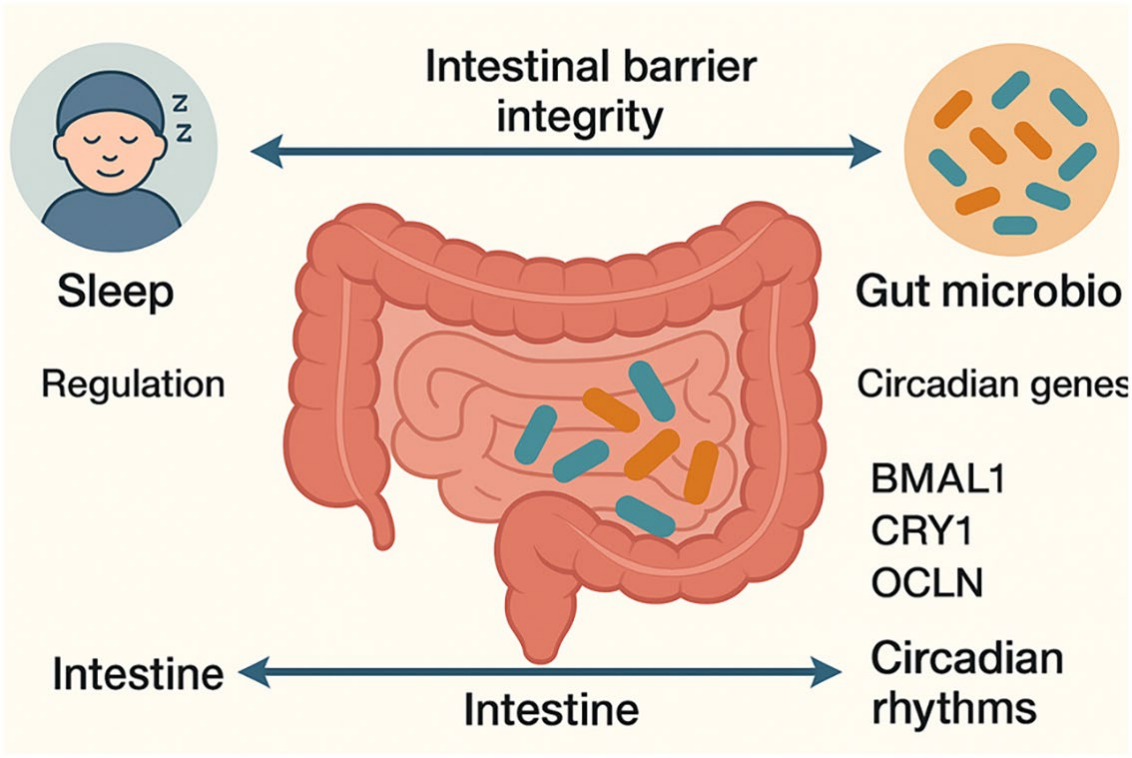
Depiction of the reciprocal relationship among sleep, the gastrointestinal system, the immune system, and microbiota. The diagram highlights how sleep regulates immune responses and gut health, while gut microbiota influence circadian rhythms, neurotransmitter production, and immune modulation. Disruptions in one system can trigger cascading effects on the others, emphasizing the integrated nature of the gut–sleep–immune axis.

Sleep disruptions induce alterations in gut microbiota that influence both its composition and functional capacity, subsequently impacting sleep quality. These changes result from host circadian rhythm gene modulation. This modulation involves the downregulation of genes including brain, BMAL1, OCLN, and CRY1. This disruption damages the intestinal barrier, reduces beneficial bacteria like lactobacilli and rumen ciliates, and promotes gut microbiota dysbiosis, affecting sleep quality (Yang et al., 2023). It is demonstrated through knockout models that the dysfunction of the central clock gene BMAL1 results in compromised gut epithelial function, microbial dysbiosis, increased susceptibility to gut infections, and disruptions in lipid metabolism (Godinho-Silva et al., 2019), highlighting the complex relationship between gut health and circadian control (Figure 4).

Smith et al. used actigraphy and gut microbiome sampling to determine the relationship between the gut microbiome and sleep physiology measurements. Taking into consideration the fact that these factors might have an effect on the link between sleep and the composition of the gut microbiome, we carried out a neurobehavioral evaluation and recorded immune system biomarkers. Total microbiome diversity increases sleep efficiency and duration, but decreases wakefulness after sleep onset. Total microbiome diversity associated positively with interleukin-6, a cytokine known to affect sleep. The richness of the Bacteroidetes and Firmicutes phyla was found to have a positive correlation with the efficiency of sleep, levels of interleukin-6, and abstract thinking, according to the findings of an analysis of the composition of individuals’ microbiomes. At the end of the day, we discovered that certain taxa, namely Lachnospiraceae, Corynebacterium, and Blautia, displayed a negative correlation with sleep metrics. The findings that we obtained establish connections between the immune system, the physiology of sleep, the composition of the gut microbiome, and cognitive processes. They may develop mechanisms to enhance sleep by modifying the gut microbiome (Smith et al., 2019).

The current study examined alterations in gut microbiota composition induced by sleep deprivation in a separate investigation and assessed whether these changes contributed causally to cognitive impairment and chronic inflammatory conditions. SD caused gut dysbiosis, inflammation, and cognitive decline. In germ-free mice (GF), gut microbiota absence reduced sleep deprivation-induced inflammation and cognitive deficits. Over the course of the process of transplanting the “SD microbiota” into germ-free mice, it was observed that the TLR 4/NF-κB signaling pathway was activated. This activation led to a decline in cognitive function that was experienced by the mice that received the transplant. In both the medial prefrontal cortex and the hippocampus, mice that possessed “SD microbiota” displayed increased levels of microglial activity and neuroinflammation. The findings indicate that gut dysbiosis exacerbates sleep deprivation-induced cognitive impairments and central and peripheral inflammatory processes, potentially leading to avenues for treatments to alleviate the adverse effects of sleep deprivation (Wang et al., 2021).

A study comparing patients with narcolepsy type 1 to a healthy control group revealed differences in the abundance of taxonomic units within the Bacteroides, Flavonifractor, and Bacteroidetes families. The research was conducted to investigate differential bacterial abundance (Lecomte et al., 2020). Rats that were deprived of sleep for a period of 48 h exhibited significant compositional changes in comparison to the control group. During these changes, Butyricimonas, Butyricicoccus, Intestinimonas, Lactobacillus, and Alistipes decreased in relative abundance while Streptococcus increased. Changes in the functional characteristics of the rat gut microbiota were observed. These changes included a decrease in 17 KEGG metabolic pathways that are associated with the metabolism of amino acids, lipids, and carbohydrates, as well as an increase in two KEGG pathways that are associated with the production of LPS. After a week of sleep recovery, these changes were found to be reversible (Wang et al., 2022c).

Furthermore, excessive expression of the circadian rhythm gene CRY1 has shown promise in reducing inflammation brought on by lack of sleep (Qin and Deng, 2015), implying a direct involvement in sleep control. On the other hand, sleep problems can cause CRY1 down-regulation as well as intestinal mucosal damage. OCLN, on the other hand, demonstrated to reduce insulin resistance and inflammation linked with sleeplessness (Huang et al., 2017), highlighting the potential therapeutic benefits of modulating these molecular pathways during treatment. Research indicates that alterations in the host’s sleep can modify the expression of genes associated with circadian rhythm. This can undermine the integrity of the gut barrier and disturb the balance of the gut microbiota. Conversely, alterations in gut microbiota can influence sleep quality. A comprehensive understanding of the interactions among these components is beneficial for the prevention and treatment of sleep disorders.

It is of the utmost importance to gain an understanding of the ways in which metabolites that are derived from the microbiota of the gut, like bile acids, indoles, and neurotransmitter precursors, influence circadian rhythms and sleep. This is in addition to the genetic interactions that are involved. These metabolites influence the regulation of sleep levels and the expression of circadian genes as they are signaling molecules that engage with host receptors.

This establishes a crucial basis for the formulation of innovative therapeutic approaches, as it enables one to acquire an understanding of the intricate interactions that exist between metabolite profiles, the composition of the gut microbiota, and the expression of circadian genes. The use of targeted interventions, like precision probiotics, dietary modifications aimed at increasing the production of beneficial metabolites, and pharmaceutical agents that modify specific circadian pathways, may be beneficial for individuals who suffer from sleep disorders. These interventions may improve sleep–wake cycles and health. While this evidence establishes a strong intrinsic link between the gut and sleep, lifestyle factors can significantly modulate this axis. Among these, physical exercise has emerged as a particularly potent, non-pharmacological modulator of the gut microbiome, as will be discussed next.

### 3.3 Exercise-induced modulation of gut microbiota

Initial evidence for exercise’s effect on gut microbiota emerged from cross-sectional studies, which compare different groups at a single point in time (Table 2). A seminal study by Clarke and colleagues, for instance, found that professional rugby players possessed a significantly higher gut microbial alpha diversity compared to lean, sedentary controls (Clarke et al., 2014). However, the cross-sectional design of such studies, while foundational, cannot establish causality and is highly susceptible to confounding variables. As the authors themselves noted, the athletes’ significantly higher protein consumption accounted for a substantial portion of the microbial variation, making it difficult to isolate the independent effect of exercise from diet.

**Table 2:** Summary of key studies investigating the effects of exercise on the gut microbiota.

Author (s) and year	Study design	Sample/participants	Key findings related to exercise and microbiota	Limitations/key context	Reference
Clarke et al. (2014)	Cross-Sectional	40 professional rugby players vs. 46 healthy controls (matched for age & BMI)	Athletes had significantly higher microbial alpha diversity. Increased abundance of Akkermansia and SCFA-producing taxa.	Foundational but cross-sectional; cannot infer causality. Diet (high protein intake in athletes) was a major confounding factor.	Clarke et al. (2014)
Allen et al. (2018)	Controlled Longitudinal	32 previously sedentary adults (18 lean, 14 obese) in a 6-week supervised exercise program followed by a 6-week washout.	Exercise increased fecal SCFA concentrations (esp. butyrate) in lean participants. Effects were dependent on obesity status.	Strong design with dietary controls. Crucially showed that most microbial changes were transient and reversed during the washout period.	Allen et al. (2018)
Cronin et al. (2018)	Longitudinal Intervention	90 overweight/obese adults in an 8-week exercise program (with/without whey protein).	Null finding: No significant changes in microbial taxonomic composition or metabolic pathways were observed in the exercise groups.	Important null result. Suggests that the exercise duration or intensity may have been insufficient to alter a less responsive microbiome in an obese cohort.	Cronin et al. (2018)
Munukka et al. (2018)	Longitudinal Intervention	17 overweight, sedentary women in a 6-week endurance exercise program.	Increased relative abundance of *Akkermansia muciniphila*. However, only ~50% of participants were “responders.”	Highlights significant inter-individual variability. Demonstrates that not everyone’s microbiome responds to the same exercise stimulus.	Munukka et al. (2018)
Zhong et al. (2021)	Randomized Controlled Trial (RCT)	28 sedentary elderly women (14 exercise, 14 control) in an 8-week program.	Exercise induced changes in OTU clustering. Increased abundance of Verrucomicrobia and decreased Proteobacteria.	RCT design is a strength. Shows that exercise can modulate the gut microbiota even in an elderly population, which is often less responsive.	Zhong et al. (2021)

Among the organisms that produce butyrate are *F. prausnitzii* and *R. hominis* (Louis and Flint, 2009), while *A. muciniphila* has been linked to a lean BMI and better metabolic health (Dao et al., 2016). Several studies have also investigated the link between cardiorespiratory fitness and microbial composition. These studies have been carried out through a variety of research methods. Durk et al. (2019) found that VtO2max has a significant correlation with the ratio of Firmicutes to Bacteroidetes, which are the two primary types of bacteria that are found in the human gut microbiota. Barton et al. (2018) metagenomic research indicates that athletes display alterations in gut microbial pathways linked to carbohydrate metabolism and amino acid biosynthesis, as well as increased levels of fecal SCFA.

The cross-sectional design of these studies, along with their failure to consider dietary influences and other variables, limited their findings related to the gut microbiota. The microbiota composition shows considerable variability among individuals; those who are physically active generally maintain different dietary patterns than their sedentary counterparts. For example, Clarke et al. (2014) found that a significant number of the observed variations in the gut flora could be attributed to the increased consumption of protein among elite rugby players.

To address the limitations of cross-sectional designs, controlled longitudinal studies were essential. A key study by Allen et al. represented a significant methodological step forward by subjecting sedentary individuals to a six-week supervised exercise program with strict dietary controls (Allen et al., 2018). This design allowed for the demonstration of a direct, diet-independent effect of exercise on the microbiota, including an increase in butyrate-producing taxa in lean participants. However, the study also revealed a critical limitation: the majority of these positive microbial shifts were reversed after a subsequent six-week sedentary washout period. This finding is crucial as it suggests that the benefits of exercise on the gut microbiome are transient and require sustained, ongoing physical activity to be maintained.

However, the effects of exercise are not always robust, highlighting potential conflicts in the literature. For example, a study by Cronin et al. involving overweight and obese adults found that an eight-week program of resistance and aerobic training failed to induce significant changes in gut microbial taxonomic composition or metabolic pathways (Cronin et al., 2018). This null result is important as it contrasts with other findings and suggests the existence of a therapeutic threshold. The lack of a strong microbial response may be due to several factors, including an insufficient exercise duration or intensity to overcome the microbial inertia often seen in obese individuals. Furthermore, the reliance on self-reported diet may have masked confounding variables, underscoring the necessity of rigorous controls in intervention studies.

Munukka et al. (2018) investigated whether or not endurance exercise had an effect on the gut metagenome in seventeen women who were overweight and sedentary. Over the course of a period of 6 weeks, cycling at a light to moderate intensity led to a decrease in the number of Proteobacteria and an increase in the relative abundance of *A. muciniphila*. Approximately 50 % of the participants’ microbiomes exhibited a notable response to the activity, representing one of the most intriguing findings. Crucially, this also means half of the cohort were ‘non-responders,’ which highlights the significant inter-individual variability discussed in Section 6.3. This inconclusive result underscores that exercise is not a universal driver of microbial change and suggests that host factors like baseline microbiome composition or genetics likely mediate the response. Exercise training was found to reduce the prevalence of a number of genes that are linked with the metabolism of amino acids and fructose, according to studies published in the field of metagenomics.

Although prolonged or more vigorous aerobic exercise may be necessary to effect alterations in taxonomic and metagenomic profiles, these results suggest that exercise independently influences the composition of gut flora. It’s possible that the microbiota of people who are lean respond better to exercise than the microbiota of people who are overweight or obese. Exercise has the potential to change the gut flora in a number of different ways. Exercise may also damage the gut mucus layer, which prevents mucosa-associated bacteria like *A. muciniphila* from attaching to the gut epithelium by providing a substrate. Exercise, especially when performed for extended durations or in elevated temperatures, elevates core temperature and induces heat stress (Rowell et al., 1968). High-intensity exercise reduces intestinal blood flow by over 50% in 10 min, causing gut ischemia (van Wijck et al., 2011). Reperfusion of the splanchnic bed is accelerated by resting. In spite of the fact that the intestine is anaerobic, the epithelial cells that line the gut primarily practice oxidative metabolism. High-intensity exercise has the potential to temporarily impair the function of the gut barrier (van Wijck et al., 2011; Otte et al., 1985). Exercise-induced heat stress and ischemia can affect gut microorganisms by temporarily increasing direct interaction between the gut mucosal immune system and gut lumen and mucosa bacteria.

Regular exercise may reduce microorganism-immune system contact at rest, even though acute exercise temporarily increases intestinal permeability. There has been a correlation established between the presence of higher HSPs in the gut and the maintenance of tight junction protein degradation between epithelial cells (Dokladny et al., 2006). Thus, exercise may be a gut hormetic stressor that promotes good adaptations and gut barrier resilience.

This study investigates the impact of an 8-week exercise program on gut microbiota in sedentary elderly women, as reported by Zhong et al. (2021) 14 women were randomly assigned to either the control group or the exercise group. Through repeated-measures analysis of variance, gut microbiota changed. No significant alpha diversity change occurred. After intervention, operational taxonomic units (OTUs) formed two clusters. Fusobacteria, Betaproteobacteria, and Bifidobacteriales distinguished themselves at the phylum, class, and order levels (*F* = 5.257, *p* = 0.045, *F* = 5.149, *p* = 0.047, and *F* = 7.624, *p* = 0.020). Significant interaction occurred between two Actinobacteria groups (*F* = 8.434, *p* = 0.016). The main effect of groups at the family and genus levels was significant for Bifidobacteriaceae, Bifidobacterium, and Gemmiger. The results suggest that sedentary older women may change their gut microbiota abundance and OTU clustering after 8 weeks of exercise. Exercise may increase Verrucomicrobia and decrease Proteobacteria (Zhong et al., 2021).

Numerous studies have evidenced the correlation between physical activity (PA) and the regulation of the gastrointestinal microbiome (GM). The influence of various physical activities, physical education, training methods, and age-related factors on human GM remains unclear. This systematic review aims to evaluate and compile the latest scientific data regarding the bidirectional interaction between physical activity/physical exercise and the human gut microbiome. The review will focus on the different types of PA/PE and their age-related effects in both healthy and unhealthy individuals. Systematic searches were conducted using internet search engines including Medline (PubMed), Google Scholar, Web of Science, and the Cochrane Library. The PICOS format, encompassing populations, intervention, exposure, and outcomes, was employed for the extractions. The Oxford Quality Scoring System Scale, JBI Critical Appraisal Checklist for Analytical Cross-Sectional Studies, and ROBINS-I checklist were employed for qualitative evaluation of the review. Protocol registered with PROSPERO: CRD42022302725. The information extracted encompasses the author, year of publication, study design, participant demographics, type of physical activity or education, protocol/workload, dietary assessment, intervention duration, measurement tools, and primary outcomes. Subsequent to screening, only 76 full texts were analyzed from a total of 694 abstracts reviewed by two authors from the team. Ultimately, twenty-five research papers were accepted. These findings show that aerobic exercise increases GM diversity, resistance training decreases it, Prevotella genus abundance is related to training duration, and GM richness and diversity do not change at the WHO minimum dose. Physical activity increases pro-inflammatory bacteria, but PA does not affect GM genetic diversity in older adults (60+). Diet control, sequencing methods, and study training parameter heterogeneity are the biggest confounding factors. This systematic review can examine the relationship between PA/PE and intestinal microbiota and provide advice on athletics and health management (Cataldi et al., 2022).

Another way exercise could affect the gut microbiome is by changing gut motility or ENS activity. Exercise has been demonstrated to speed up gas movement across the digestive (GI) tract and to lower transit time in the large intestine (Dainese et al., 2004; Song et al., 2012). Exercise is known to raise sympathetic and vagal tone in the autonomic nervous system (Freeman et al., 2006). The effect on the complex neuronal network of the gut remains ambiguous. Changes in gastrointestinal transit, whether regional or global, can significantly influence intestinal pH, biofilm formation, mucus production, and the availability of nutrients for bacteria. Aerobic exercises generally enhance abdominal mechanical forces, potentially influencing gut mobility or intestinal mixing.

Training in exercise might also change the enterohepatic flow of bile acids. Meissner et al. (2011) hypercholesterolemic mice given a running wheel for 12 weeks secreted and excreted more bile acid than sedentary mice. The deficiency of bile acids is linked to significant changes in the communities of microorganisms that live in the gut, which is commonly referred to as gut dysbiosis (Kakiyama et al., 2013). Consequently, Changes in the bile acid pool may substantially influence the gut microbiota and its functionality.

Last but not least, physical activity requires the contraction of skeletal muscles, which causes a significant alteration in metabolic flux, which is the rate at which molecules move through metabolic pathways. The alteration in metabolic flux results in the secretion of neuroendocrine hormones, myokines, and metabolites, which may engage with the gut directly or indirectly through a shared interface with the immune system (Egan and Zierath, 2013). Exercise leads to significant lactate release into the bloodstream, potentially influencing the pH of the intestinal lumen if lactate is secreted into the gut lumen. Further research is necessary to identify the mechanisms by which gut microbiota adapts to exercise training. Table 3 delineates significant alterations in gut microbiota phyla resulting from exercise. Having established that the gut-sleep axis is deeply interconnected (Section 3) and that exercise is a powerful modulator of the gut microbiome (Section 4), the central question becomes: what are the precise biological mechanisms that connect all three domains? The following section will synthesize the evidence by focusing on the shared physiological pathways—inflammation, microbial metabolites, and neuroendocrine signaling—that constitute this tripartite relationship.

**Table 3:** Exercise-induced changes in major gut microbiota phyla.

Phylum	Exercise-associated changes	References
Firmicutes	↑↑ SCFA-producing bacteria (Ruminococcaceae, Lachnospiraceae, Erysipelotrichaceae); *Faecalibacterium prausnitzii*, Dorea, ↑ Coprococcus, Roseburia; Veillonella linked to improved performance	Estaki et al. (2016), Munukka et al. (2018), Zhong et al. (2021), Cataldi et al. (2022), Nylund et al. (2015), Palmnäs-Bédard et al. (2022), Morishima et al. (2021), Cheng et al. (2022), Samuel and Gordon (2006), Bielik et al. (2022), Verheggen et al. (2021), Shukla et al. (2024), Huang et al. (2024)
Bacteroidetes	↑ *Prevotella intermedia*, *Bacteroides caccae*, Parabacteroides with high-intensity exercise and sustained routines	Cataldi et al. (2022), Bielik et al. (2022), Liang et al. (2019), Warbeck et al. (2021), Zafar and Saier (2021)
Verrucomicrobia	↑ *Akkermansia muciniphila*; associated with metabolic protection and anti-inflammatory effects	Bressa et al. (2017), Zhong et al. (2021), Nylund et al. (2015), Palmnäs-Bédard et al. (2022), Torquati et al. (2023), Barton et al. (2021), Liu et al. (2020), Alam et al. (2022), Keohane et al. (2019), Vacca et al. (2020)
Actinobacteria	↑ Bifidobacterium; produces acetate and lactate; supports gut barrier and SCFA cross-feeding	Bressa et al. (2017), Torquati et al. (2023), Zhao et al. (2024), Fusco et al. (2023), Goto (2019), Zhu et al. (2020), Castro-Mejía et al. (2020), Binda et al. (2018), Magzal et al. (2022), O'Donovan et al. (2020)
Proteobacteria	↑↓Proteobacteria with moderate exercise; in elite athletes post-intense training; includes pro-inflammatory *Escherichia coli*	Bressa et al. (2017), Munukka et al. (2018), Zhong et al. (2021), Zhu et al. (2020), Jang et al. (2019), Quiroga et al. (2020), Lkhagva et al. (2021), Han et al. (2020), Tabone et al. (2021), Donati Zeppa et al. (2021)

## 4 Shared mechanistic pathways: integrating exercise, microbiota, and sleep

### 4.1 The inflammatory pathway as a common mediator

Systemic low-grade inflammation serves as a critical mechanistic node where sleep, gut health, and exercise intersect. A pathological feedback loop can be established where gut dysbiosis and poor sleep fuel inflammation, which in turn can further disrupt sleep architecture. Exercise provides a powerful counter-regulatory mechanism by directly targeting this inflammatory cycle.

Immune dysfunction is a key consequence of sleep disorders. Research shows that sleep deprivation can lead to intestinal barrier damage and increased translocation of bacterial lipopolysaccharide (LPS), which triggers inflammatory responses via the NF-κB and TLR4 pathways (Wang et al., 2021). This state is characterized by a disrupted balance of cytokines, with a significant increase in pro-inflammatory factors such as IL-1β, TNF-*α*, and IL-6 (Fang et al., 2023). Clinically, individuals with insomnia exhibit higher circulating levels of IL-6, a cytokine that is itself known to affect sleep (Smith et al., 2019; Burgos et al., 2006; Hogan et al., 2003).

Moreover, studies highlight the close relationship between sleep quality and immunological elements like TNF-\u03b2, IL-1, and IL-10, stressing their control functions (Kapás and Krueger, 1992; Toth and Opp, 2001). The findings highlight a mutual relationship between the sleep and gut microbiome, facilitated by intricate immune regulatory mechanisms. This interaction highlights the significance of the gut-sleep-health axis.

The gut-associated lymphoid tissue, which can be found in both the small and large intestines, is responsible for the storage of 70 % of the immune cells that are distributed throughout the body. Hoffman-Goetz et al. (2010), Hoffman-Goetz et al. (2009), and Packer and Hoffman-Goetz (2012) proved that exercise downregulates pro-inflammatory cytokines and upregulates antioxidant and anti-inflammatory enzymes in intraepithelial lymphocytes. Immune cells near bacteria produce antibacterial chemicals needed for host-microbial homeostasis (Ismail et al., 2011). At rest, trained athletes have lower bacterial endotoxin lipopolysaccharide levels than sedentary people (Lira et al., 2010) as well as an increased HSP reaction to heat stress (Fehrenbach et al., 1985).

### 4.2 Microbial metabolites: SCFAs as system-wide messengers

Beyond inflammatory signaling, the gut microbiota communicates with the brain via the production of neuroactive metabolites. Short-chain fatty acids (SCFAs)—primarily acetate, propionate, and butyrate—are produced by microbial fermentation of dietary fiber and function as crucial signaling molecules within the gut-brain axis (Zhao N. et al., 2022). These metabolites have been directly implicated in sleep regulation. Of these, butyrate is particularly noteworthy, as direct administration has been shown to enhance non-REM sleep and reduce sleep fragmentation (Lively and Schlichter, 2018; Liu et al., 2021; Zhao N. et al., 2022; Gao et al., 2019; Wang et al., 2023), while disruptions in SCFA production are linked to sleep disturbances and inflammation (Lively and Schlichter, 2018; Morrow and Opp, 2005). More recently, Bressa et al. (2017) *Roseburia hominis*, *Faecalibacterium prausnitzii*, and *Akkermansia muciniphila* were found to be present in higher concentrations in women who were physically active and exercised for a minimum of 3 h for each week. Estaki et al. (2016) younger people’s cardiorespiratory fitness was positively linked with microbial diversity and butyrate-producing bacteria. Nevertheless, the number of butyrate-producing taxa as well as the levels of faecal acetate and butyrate were increased over the course of 6 weeks of exercise in individuals who were lean. This evidence allows for the formulation of a clear, testable hypothesis: regular exercise remodels the gut microbiome to favor butyrate production, and this increase in circulating butyrate acts as a key signaling molecule at the central nervous system to directly improve sleep quality and architecture.

### 4.3 Neuroendocrine and neurotransmitter regulation

The third key mechanistic link involves the regulation of the hypothalamic–pituitary–adrenal (HPA) axis and the synthesis of key neurotransmitters. The HPA axis is the body’s primary stress response system, culminating in the release of cortisol, a hormone that is fundamentally linked to arousal and wakefulness (Bi et al., 2022; Keller et al., 2017). Chronic stress, poor sleep, and gut dysbiosis can all lead to HPA axis hyperactivity. Gut dysbiosis, in particular, can activate the HPA axis, leading to elevated cortisol levels which disrupt the natural sleep–wake cycle (Burokas et al., 2017).

Simultaneously, the gut microbiota plays a direct role in synthesizing or modulating neurotransmitters essential for sleep and relaxation, including serotonin and GABA. Gut microbes metabolize dietary tryptophan into serotonin, the precursor to melatonin, and certain species, such as *Lactobacillus* and *Bifidobacterium*, can produce GABA, the primary inhibitory neurotransmitter in the brain (Zhang et al., 2021; Roth et al., 2021; Bravo et al., 2011). Exercise is a well-established, non-pharmacological regulator of the HPA axis, known to buffer the physiological response to stress. By promoting a more balanced gut microbiome, exercise may therefore provide a dual benefit: it helps normalize HPA axis activity while supporting the microbial communities that produce sleep-promoting neurotransmitters, thus creating a more favorable neurochemical environment for restful sleep. While this mechanistic framework provides a compelling model for the exercise-microbiota-sleep axis, it is crucial to critically evaluate the strength and limitations of the evidence upon which it is built.

## 5 Critical appraisal, clinical applications, and future directions

While the evidence for an exercise-microbiota-sleep axis is compelling, the field is in its early stages and relies heavily on correlational findings. A critical appraisal reveals several unresolved questions and controversies that must be addressed to move the field forward.

### 5.1 The challenge of causality

The primary unresolved issue is establishing causality. Does exercise improve sleep *via* the microbiome, or are these parallel, independent benefits of a healthy lifestyle? Most human studies are observational and cannot dissect this relationship. Future research must incorporate designs capable of testing for mediation. For example, studies in animal models could compare the effects of exercise in germ-free, antibiotic-treated, and conventionally-colonized subjects to isolate the microbiota’s causal role.

### 5.2 Defining the optimal “exercise prescription”

A significant knowledge gap exists regarding the optimal exercise parameters. While moderate aerobic exercise appears favorable, high-intensity exercise can acutely increase intestinal permeability. Does resistance training confer similar benefits to aerobic exercise on the microbiota relevant to sleep? Answering these questions is critical for developing effective, evidence-based recommendations.

### 5.3 The confounding roles of diet and individual variability

The high inter-individual variability in microbial responses to exercise presents a major challenge. As noted by Clarke et al. (2014), diet can be a powerful confounder that explains a significant portion of microbial variation. Future studies must implement rigorous dietary controls to isolate the independent effect of exercise. This variability suggests that a ‘one-size-fits-all’ approach is unlikely to be effective, pointing toward a future of personalized, microbiota-informed interventions.

### 5.4 Population-specific applications and integrated interventions

The evidence synthesized in this review points toward significant therapeutic potential, particularly for specific populations. For example, in cancer survivors, exercise-mediated gut microbiome support may improve body composition and cognitive function (Matei et al., 2023). Similarly, targeting this triadic relationship could offer new strategies for individuals with neurodegenerative diseases (Rojas-Valverde et al., 2023) or children with obstructive sleep apnea syndrome (OSAS), where microbial profiles are already linked to inflammatory markers (Huang et al., 2024).

Ultimately, the interaction among these systems suggests that integrated strategies will be most effective. Rather than addressing each factor in isolation, comprehensive health plans that combine regular physical activity, dietary interventions to support gut health, and sleep hygiene practices may yield the greatest benefits. Future data-driven health coaching that incorporates multi-omics profiles with real-time monitoring of activity and sleep could provide highly personalized recommendations to optimize these interconnected systems for health and disease prevention (Marabita et al., 2022).

## 6 Conclusion

The evidence reviewed in this paper supports the existence of an integrated exercise-microbiota-sleep axis, where each component dynamically influences the others. We have moved beyond a descriptive summary to propose that this tripartite relationship is governed by concrete biological mechanisms, primarily shared inflammatory pathways, the production of neuroactive microbial metabolites like SCFAs, and the regulation of the neuroendocrine stress response.

Regular physical activity emerges not just as a behavioral intervention for better sleep, but as a powerful modulator of the gut ecosystem. By promoting an anti-inflammatory environment and enriching for beneficial, metabolite-producing bacteria, exercise appears to directly target the root causes of gut-brain signaling disruptions that can impair sleep. However, as our critical appraisal highlights, the field is nascent. Future research must prioritize studies that can establish causality, define optimal exercise prescriptions, and account for individual variability. The conceptual framework presented in this review and illustrated in Figure 1 offers a roadmap for this research, suggesting that targeting the gut microbiota through personalized exercise and lifestyle interventions represents a highly promising, non-pharmacological strategy for enhancing sleep quality and overall health. Future research employing longitudinal designs and multi-omics approaches is essential to translate these findings into targeted, personalized lifestyle interventions for enhancing sleep quality and overall health.
